# Downscaled gridded global dataset for gross domestic product (GDP) per capita PPP over 1990–2022

**DOI:** 10.1038/s41597-025-04487-x

**Published:** 2025-01-30

**Authors:** Matti Kummu, Maria Kosonen, Sina Masoumzadeh Sayyar

**Affiliations:** Water and Development Research Group, Aalto University, Espoo, Finland

**Keywords:** Economics, Geography

## Abstract

We present a comprehensive gridded GDP per capita dataset downscaled to the admin 2 level (43,501 units) covering 1990–2022. It updates existing outdated datasets, which use reported subnational data only up to 2010. Our dataset, which is based on reported subnational GDP per capita data from 89 countries and 2,708 administrative units, employs various novel methods for extrapolation and downscaling. Downscaling with machine learning algorithms showed high performance (R^2^ = 0.79 for cross-validation, R^2^ = 0.80 for the test dataset) and accuracy against reported datasets (Pearson R = 0.88). The dataset includes reported and downscaled annual data (1990–2022) for three administrative levels: 0 (national; reported data for 237 administrative units), 1 (provincial; reported data for 2,708 administrative units for 89 countries), and 2 (municipality; downscaled data for 43,501 administrative units). The dataset has a higher spatial resolution and wider temporal range than the existing data do and will thus contribute to global or regional spatial analyses such as socioenvironmental modelling and economic resilience evaluation. The data are available at 10.5281/zenodo.10976733.

## Background & Summary

The rapid increase in the availability of various global earth system and climate datasets over the past few decades^1^ has made it possible to conduct different risk and vulnerability assessments at fine spatial scales globally^2^. To understand the effects on human life, these earth system and climate data should be analysed together with long-term continuous socioeconomic datasets^3^. However, traditionally, socioeconomic data are provided on a national level, creating a mismatch in scales and averaging out within-country heterogeneity. This, in turn, might lead to high inaccuracies in the results. Over the past decade, an increasing number of subnational or gridded datasets for various socioeconomic variables have been published, including data on migration patterns^4^, urbanisation^5^, gross national income (GNI) per capita and income inequality (Gini coefficient)^6^, and the human development index (HDI)^7,8^.

One of the most commonly used indicators for economic development is gross domestic product (GDP). While there are global subnational GDP per capita (or gross regional product (GRP) per capita) datasets, they are rather old—they end in 2015 with the latest subnational data from 2010 (ref. ^8^) or for only one year, 2005 (ref. ^9^)—or they do not cover data for all countries and each year (DOSE v2 dataset^10^) or both^11^. Thus, there is no harmonised and gap-filled global subnational or gridded GDP per capita dataset that spans over recent decades. Here, we constructed a global harmonised and gap-filled GDP per capita dataset (PPP) for 1990–2022 downscaled to the admin 2 level (n = 43,501 administrative units). We provide the data both as gridded (5 arc-min resolution) and as polygon data (with administrative area names) for three administrative levels: the admin 0 level (i.e., national), the admin 1 level (where available; otherwise, the admin 0 level) (i.e., provincial), and the admin 2 level (i.e., municipality). Furthermore, we combined the downscaled product with population count data and estimated total GDP (PPP) for each grid cell at three resolutions: 30 arc-sec (for every 5 years), 5 arc-min (for each year), and 30 arc-min (for each year).

This dataset builds on the existing subnational GDP per capita (PPP) dataset by Kummu *et al*.^8^. However, here, we introduce several updates in terms of input data as well as methodological developments. First, while the subnational data used for Kummu *et al*.^8^ are available only until 2010 (the dataset spans 2015, but the admin 1 level distribution of GDP per capita is based on up to 2010, while national data are available until 2015), here, the admin 1 level data expand until 2021, and the admin 0 level data expand until 2022. We also found data for both the admin 0 and 1 level data for more countries—covering the admin 1 level data for 89 countries (2,708 subnational units) and the admin 0 level data for 237 countries—outperforming the existing datasets, as shown in Table 1. Finally, while in Kummu *et al*.^8^, the finest scale was the admin 1 level, here, we downscaled the data to the admin 2 level. Wang and Sun^9^ also downscaled their data (to the gridded level), but their reported subnational data are based on rather few countries (Table 1), of which most are OECD countries, and only one year (2005).

**Table 1:** Comparison of existing global subnational GDP per capita products with this study.

Study	admin 0 level areas	admin 1 level areas	admin 2 level areas	Temporal coverage
This study	Reported data for 237 countries	Reported data for 2708 admin units, for 89 countries	Downscaled to 43,501 admin units; for 237 countries	1990–2022 (reported subnational data 1990–2021)
**Admin 0 level existing datasets**				
World Bank	Reported data for 194 countries	NA	NA	1960–2023
IMF (International Monetary Fund)	Reported data for 195 countries	NA	NA	1980–2023
**Admin 1 level existing datasets**				
Kummu *et al*.^8^	Reported data for 231 countries	Reported data for 1549 admin units, for 83 countries	NA	1990–2015 (subnational data 1990–2010)
DOSE v2^10^	NA	Reported data for 1,661 subnational units, across 83 countries (not full coverage for all countries)	NA	1953–2020
Wang and Sun^9^	Reported data for 197 countries	Reported data for ca. 800 subnational units, for 48 countries	NA, but downscaled to gridded level	Year 2005

We developed methods for multiple fronts, as briefly described below (see Methods for a more detailed explanation):

- We used a novel way of extrapolating missing data at the admin 0 level (the same method is also used in another article of ours, Chrisendo *et al*.^12^). While previously regional trends were used for extrapolation^8^, here, we fitted the available data of a country in question with countries with full or nearly full data extent via linear regression. We then selected the geographically closest country from among the best seven fits and used that regression model to estimate the missing years from the beginning and/or end of the time series.
- At the admin 1 level, we first calculated the ‘admin 1 level/admin 0 level’ ratio for the observed values (here called the subnational GDP ratio). The admin 0 level value, for each year for which data were available in a country in question, was estimated as the population weighted mean. Only after that we interpolated the values between years with reported data (for the missing data at the beginning and end of the study period, the last reported subnational GDP ratio was used). In this way, we were able to combine data from different sources, and it did not matter whether the original data were in local currency or USD. Only at the very end we multiplied the subnational GDP ratio with the gap-filled admin 0 level data of the country in question.
- We developed a novel way to downscale the admin 1 level data (admin 1 level where available, and elsewhere admin 0 level data) to the admin 2 level data using independent datasets (such as urbanisation level, travel time to closest city and income inequality) to train a set of machine learning models in which we used boosted ensembled trees with very good model performance (R^2^ = 0.79 for cross-validation and R^2^ = 0.80 for the testing set of the data).

Finally, while existing GDP per capita datasets provide data only at the admin 1 level^8^ or admin 1 level and gridded scale^9^, here, we provide data for three levels: the admin 0, admin 1, and admin 2 levels. For all these levels, both gridded and polygon data are shared.

Our dataset thus provides more up-to-date and finer-scale global GDP per capita PPP (and total GDP PPP) data with longer temporal resolutions than the existing datasets do. It can be used for various global or regional analyses, covering topics such as climate change impacts and associated risks^13^, exposure to natural hazards^14,15^, urban development and urbanisation patterns^16^, biodiversity conservation and species invasion^17^, economic growth, inequality^18^, and sustainable development^19^. Furthermore, our data – gaps filled over time and downscaled to the admin 2 level – might be particularly useful in data-scarce regions where high-resolution data are not available.

## Methods

**Fig. 1:**
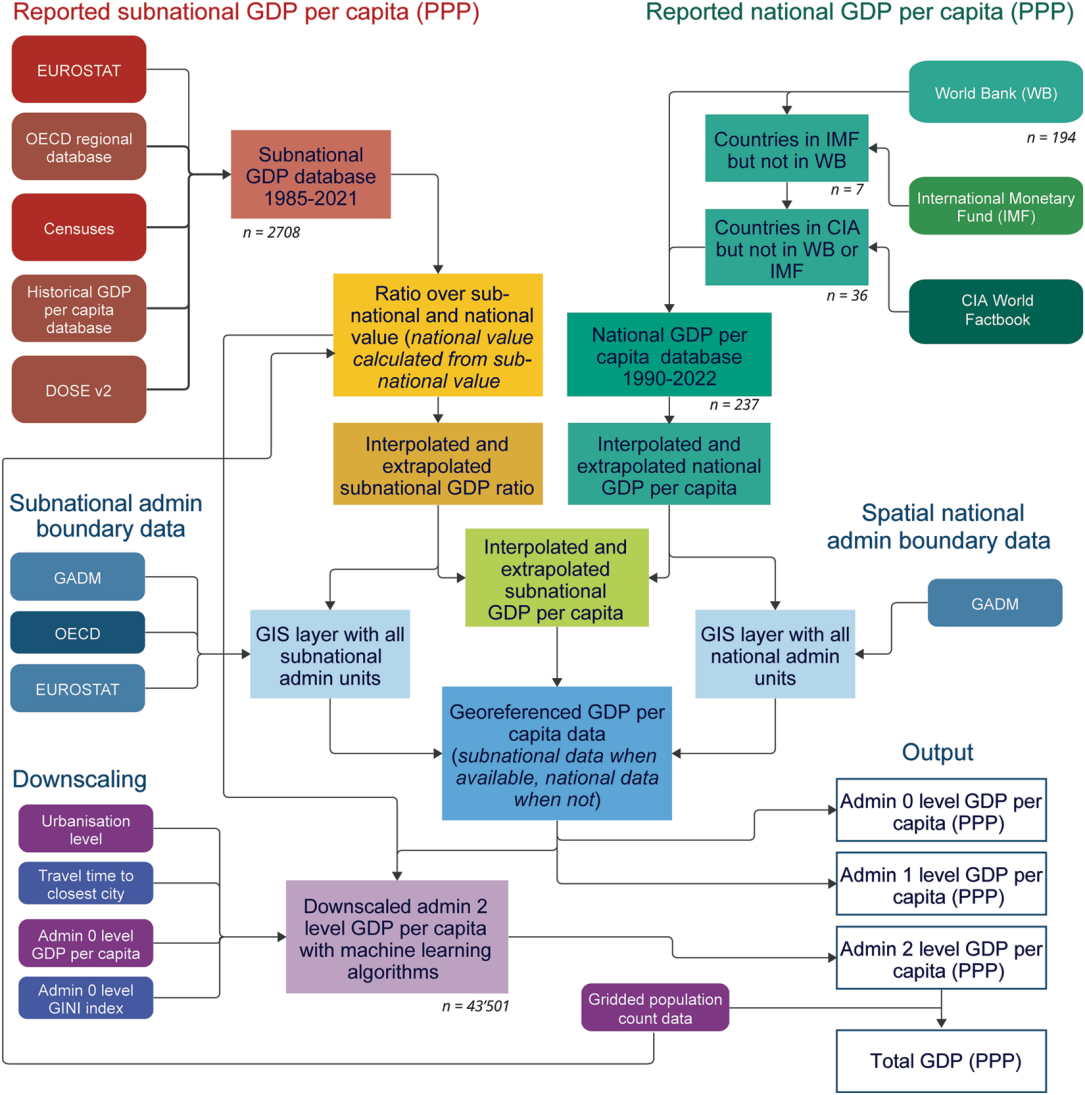
Flow diagram of the study.

We first collected admin 0 level (i.e., national) GDP per capita at purchasing power parity (PPP) data from various sources, and the gaps for missing years were filled via linear interpolation and a novel extrapolation method (see Section 2.1). We then collected admin 1 level (i.e., provincial) data from sources such as the OECD, Eurostat, and national censuses. We calculated the ratio between the admin 1 level value and the admin 0 level value (estimated from the subnational values with population data as a weight) and calculated the subnational GDP ratio (ratio over the admin 1 level value and calculated the national value). We then interpolated the subnational GDP ratio to fill the gaps between years with reported values and used the latest subnational GDP ratio for the tail and leading missing values (see Section 2.2). These subnational GDP ratios were then used with the reported admin 0 level data to estimate the GDP per capita (PPP) value at the admin 1 level. Finally, we used the subnational GDP ratio to train a here developed downscaling method based on machine learning algorithms to downscale the ratio from the admin 1 level to the admin 2 level (see Section 2.3). The overall workflow is illustrated in Fig. 1, and a more detailed explanation is given below. We used R (version 4.3.2) to conduct the study, except for downscaling, which was performed using MATLAB (version 2024a).

### Gap-filled national (admin0 level) dataset

We first collected admin 0 level (national) GDP per capita (PPP) data for 1985–2022. We combined several databases, including the World Bank^20^, the International Monetary Fund (IMF)^21^, and the data based on the Central Intelligent Agency (CIA), accessed through IndexMundi^22^, with the preference of this order (i.e., if no data were available from the World Bank, then we used the IMF, and if they were not available, we turned to the CIA). We were able to collect data for 237 nations or sovereign states, making this the most comprehensive national GDP per capita PPP dataset available. The data are in 2017 international USD.

Countries had missing data for varying numbers of years in these datasets. To fill these gaps, we used a novel methodology for filling in the missing values (also used in another article by the lead author, i.e., Chrisendo *et al*.^12^), considerably improving the existing methods by, for example, Kummu *et al*.^8^, who used regional trends for extrapolation. In the updated method, we first used linear interpolation to fill the gaps between reported values. This was performed with the na.approx function in R (under the zoo package^23^). In general, there were not many ‘holes’ between the reported years. However, a larger issue was missing values at the beginning or at the end of the time series of a country. We thus developed a multistep extrapolation method to fill in these missing values:

1. We first divided the countries into the following groups, based on the data coverage over the study period 1990–2022: a) full data extent, b) nearly full data extent (max three years missing from beginning or end), c) limited data extent (more than three years missing from either end but more than 5 data points), and d) very limited data extent (fewer than 5 data points).
2. We went through the nearly full data extent data country by country and extrapolated those using the full data extent countries. This was done by constructing a linear model (lm) between a country with missing values (targetCountry) and each country with a full extent. Next, we filtered out seven countries with the best fits based on R^2^ and from these, we chose geographically the closest country (bestClosestCountry) to the targetCountry using the centroids of each country and the distance between them. lm was then used to estimate the full time series (lmTimeseries) for a targetCountry using the data from bestClosestCountry as an input. Finally, we used the first and last reported values of the targetCountry and the corresponding values from the lmTimeseries to calculate the ratio. We used these ratios to scale the lmTimeseries to fill in the missing values from the beginning (ratio over the first nonmissing value) and end (ratio over the last nonmissing value) of the study period.
3. We combined the full dataset with the filled near-full dataset to create a combined full_nearlyFull dataset.
4. We then filled in the missing data points for the limited data extent using the full_nearlyFull dataset and the same method as in step 2.
5. We filled the countries with fewer than 5 data points by first identifying the closest country within the full_nearlyFull dataset for each country in this group (closestCountry). We then scaled the leading and trailing missing values based on the trajectory of the closestCountry in a similar way to point step 2, i.e., we calculated the ratio between the first nonmissing value and the corresponding closestCountry value. We used this to estimate the leading missing values and the ratio between the last nonmissing value and the corresponding closestCountry value. These ratios were then combined with full data series from the closestCountry to estimate the missing values.
6. Finally, we combined the full and filled datasets to create a complete dataset.

### Subnational-level dataset

We collected reported subnational-level GDP per capita (PPP) datasets from various sources. We started from the historical subnational database of Gennaioli *et al*.^11^, on which the existing subnational GDP dataset^8^ is based. We then updated subnational data for nearly every country for which subnational data were available, resulting in data for 2708 subnational units. Moreover, we expanded the temporal coverage of subnational units from 1990–2010 to 1990–2021. We used data from existing databases, such as OECD^24^ and Eurostat^25^, DOSE v2 (ref. ^10^), and national censuses. The source for each dataset used with the years it covers is given in the Supplementary Data File. For some countries, we combined data from several different sources (for different years); each source is given with the corresponding temporal coverage.

**Fig. 2:**
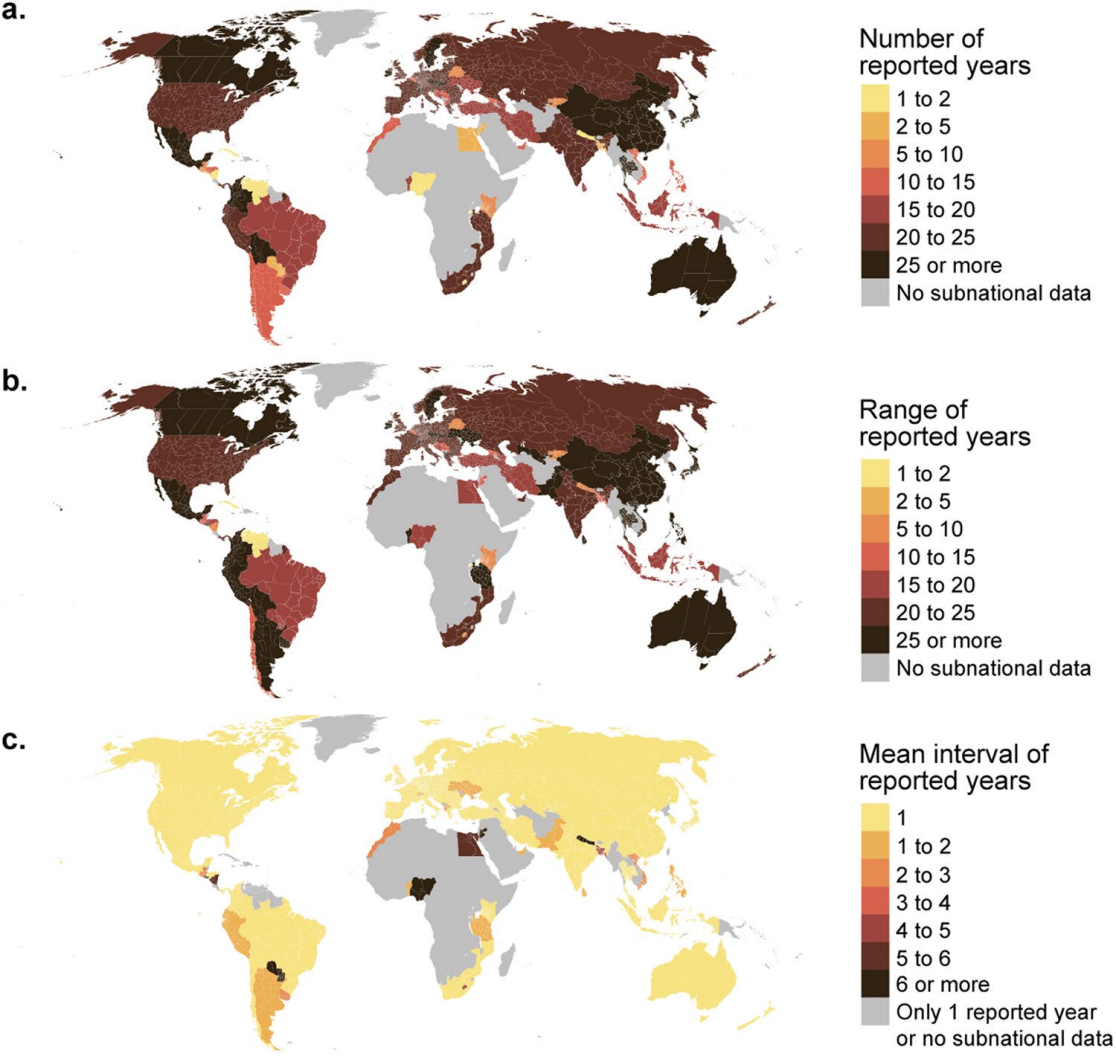
Subnational data availability over time and space. (**a**) Number of reported years (i.e., if there were data for 2000, 2005, and 2010, then the number of reported years would be 3) for each administrative area; (**b**) range of reported years (i.e., if there were data for 2000, 2005, and 2010, then the range would be 11 years) for each administrative area; and (**c**) mean interval of reported years (i.e., if there were data for 2000, 2005, and 2010, then the mean interval would be 5 years).

The number of available years with data varied greatly between the countries (Fig. 2a), as did the range of years (Fig. 2b). However, the mean interval was for most of the countries (56/89) 1, meaning that there were data for every year within the range of reported years and 2 or fewer for 72/89 countries (Fig. 2c). Notably, for the African continent, the data availability is very low, whereas in all other regions, the geographical data coverage is rather good (Fig. 2).

Due to changes in administration areas within some countries—some admin 1 level areas were split or combined within the study area (we used the www.statoids.com webpage to obtain information on these changes)—we needed to estimate the values for some administrative areas for a limited number of years. These are documented in the Supplementary Data File.

Once the subnational data were collected, we calculated the population-weighted national average GDP per capita values for each country and reported year. For population data, we used GHS-POP R2023A global gridded population data^26^. The data are given every five years with a 30 arc-sec scale. We aggregated it to a 5 arc-min scale and interpolated the missing years using linear interpolation. The derived Admin 0 level GDP per capita data were then used to calculate the ratio between the Admin 1 level and Admin 0 level GDP per capita values (subnational GDP ratio). By doing so, we were able to combine the different datasets regardless of whether the data were in USD or local currency. Additionally, later in the process, when we at the very end multiplied the ratio with reported Admin 0 level data, we ensured that the national total GDP was equal to the sum of that of subnational areas; i.e., this harmonised the admin 1 level data with reported Admin 0 level data.

The subnational GDP ratios were interpolated between the years with reported values using the na.approx function in R (under the zoo package^23^). For extrapolation, we used the nearest observed value, i.e., assuming that the relationship between the subnational areas and the admin 0 level GDP per capita remained constant (i.e., the admin 0 level data were used to scale the admin 1 level data in these areas, as in Kummu *et al*.^8^). As a result, we obtained gap-filled full time series of subnational GDP ratios for all the countries with reported admin 1 level data. This was used to train the downscaling data (see next section).

### Downscaling to the admin 2 level

We downscaled (or disaggregated) the combined subnational GDP ratio (i.e., admin 1 level/admin 0 level) dataset to admin 2 level resolution using a set of machine learning algorithms. We randomly selected 50% of the years with reported admin 1 level data from each country. This resulted in *n* = 25,608 observations.

#### Independent variables

We then prepared independent datasets for downscaling (gridded urbanisation level, gridded travel time to the closest city, income inequality at the admin 0 level, and GDP at the admin 0 level). All these data were aggregated to the admin 1 level (using the administrative boundary sequestration of the reported GDP data) by calculating the population-weighted average for each administrative unit.

##### ***Urbanisation level***

The urbanisation level is mainly positively linked to GDP per capita, as shown, for example, by Chen *et al*.^27^. We developed our own urbanisation level dataset by using the same population count data as those used to estimate the admin 0 level GDP per capita from the admin 1 level data (see Section 2.2), i.e., GHS-POP R2023A^26^.

To obtain the urbanisation level, we combined the gridded population count data with the national-level urbanisation rate from the World Urbanisation Prospects (2018 revision)^28^ as follows.

1. We first estimated the annual urbanisation rate from the World urbanisation prospects data (which includes data for each five-year period) and then interpolated and extrapolated the data for each year using the same method as that used for the admin 0 level GDP per capita (see above).
2. We then used the gridded population density and count data (see above) separately for each country and year to determine the cumulative population starting from the grid cell with the lowest population density to the highest, and we calculated the cumulative percentage of the total national population.
3. We then compared this to the national urbanisation rate, i.e., if the urbanisation rate was 43% for a given year, the threshold for population density for urban areas is the one that is reached with a cumulative percentage of the total population of 1–0.43 = 0.57. This population density is assigned a value of 1.
4. Finally, we scaled the population density data so that the grids with the highest density were assigned a value of 2, those with a threshold value of 1 and those with the lowest density were assigned a value of 0. Thus, all rural areas have a value < 1, and all urban areas have a value > 1.

If we could not estimate the urbanisation level for an administrative unit in question (this was the case for some small island states and sovereign territories), we used a value of 1 (on a scale of 0 to 2).

##### ***Travel time to the closest city***

The travel time to the closest city, which is highly linked to road infrastructure, has been shown to have an impact on economic activities^29^ and thus was selected as one of the independent variables. We used raster data of travel time to the closest city of at least 50,000 people from^30^ with a 1 km resolution for 2015. We resampled it to 5 arc-min resolution so that we were able to weight it with the population dataset when aggregating it to the administrative units.

##### ***Income inequality at the admin 0 level***

We used income inequality (Gini coefficient) as a proxy for how evenly income is distributed across the country and thus was used as an indicator of potential heterogeneity in the GDP ratio between the admin 1 level units. We used the national-level Gini coefficient, compiled by authors from multiple sources, for a total of *n* = 198 countries^6^. If a country did not have data for a Gini coefficient (this was the case for some small island states and sovereign territories), we used a value of 0.5 (on a scale from 0 to 1).

##### ***GDP per capita for the admin 0 level***

This independent variable, GDP per capita (PPP) at the admin 0 level, represents the economic status of a country where the admin 1 level unit is located. The reason for adding this to the downscaling was to take into account the different dynamics of how the GDP ratio varies within a country depending on the overall economic level of the country. As we downscaled the admin 1 level/ admin 0 level ratio, these admin 0 level GDP per capita data were independent from the ratio data.

#### Downscaling procedure

**Fig. 3:**
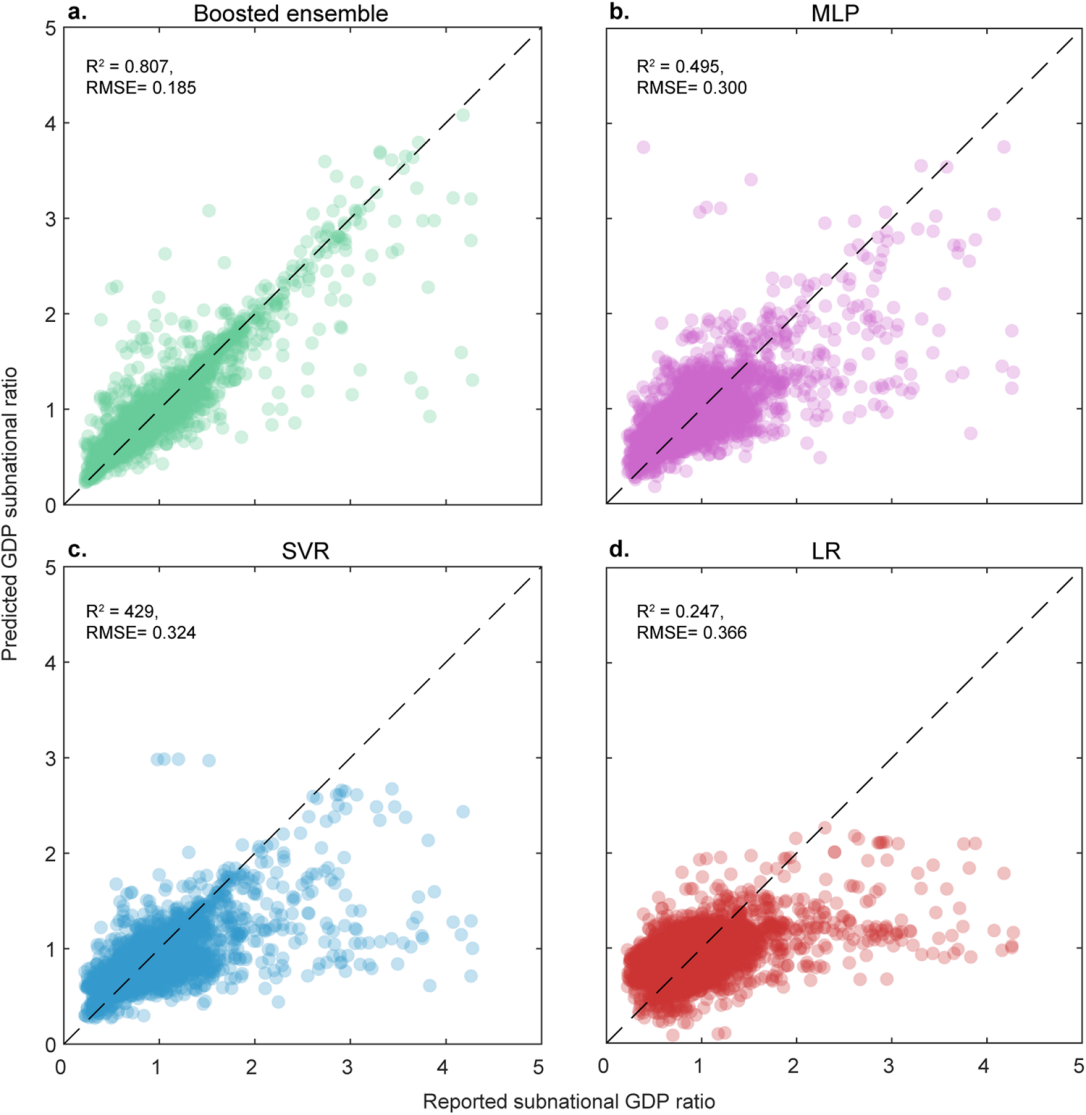
Regression plots for all downscaling models using the test dataset (20% of all data points; *n* = 5,062). (**a**) Ensemble trees model, (**b**) multilayer perceptron (MLP) model, (**c**) support vector regression (SVR) model, and (**d**) linear regression (LR) model. On the x-axis, the reported ratios over the admin 1 and admin 0 levels are shown, whereas on the y-axis, the modelled ratio is shown.

**Fig. 4:**
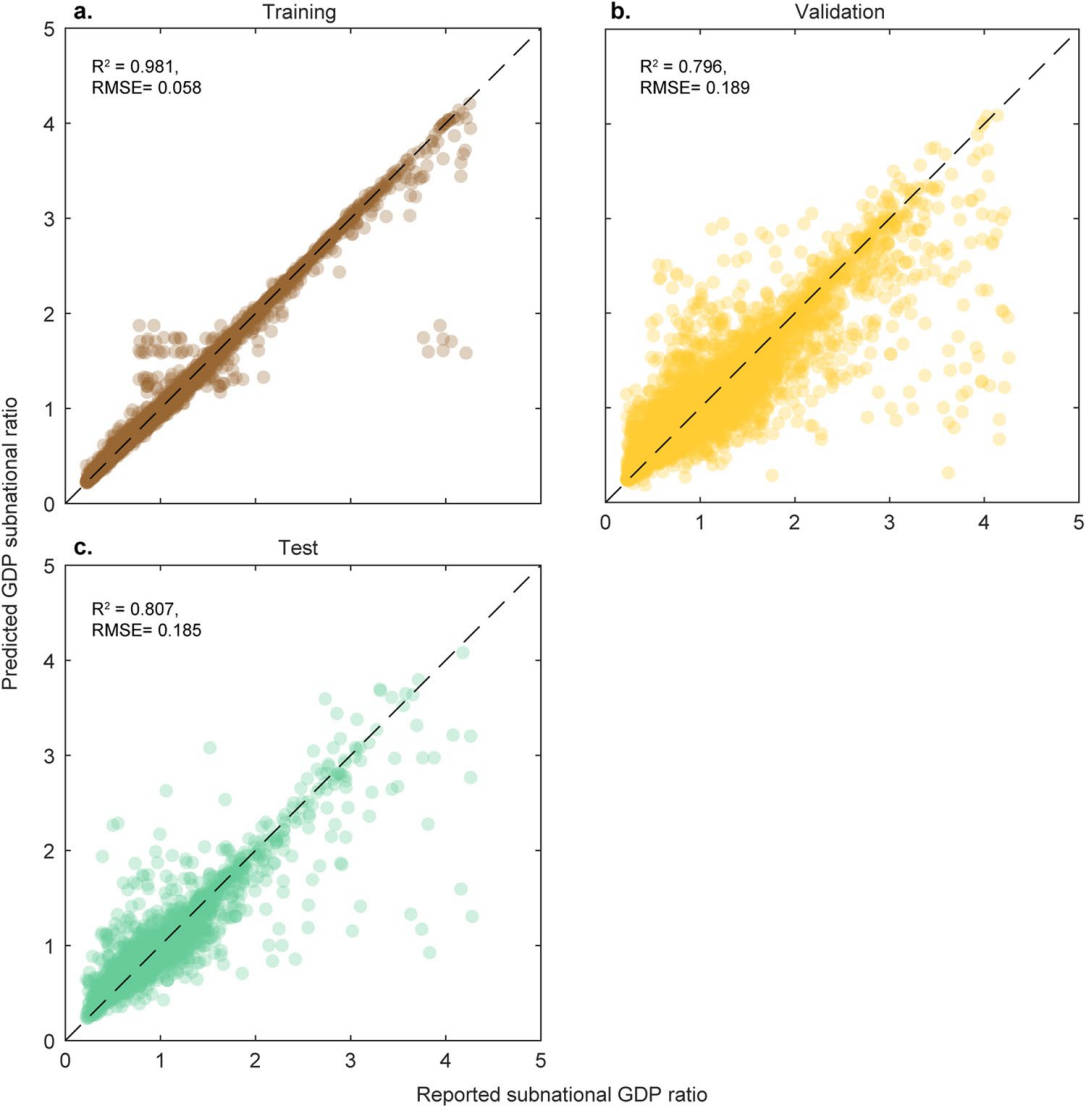
Downscaling model accuracies. Downscaling model accuracy. (**a**) for the ensemble trees model for the entire dataset (*n* = 25,307), (**b**) for the training dataset (*n* = 20,245), and (**c**) for the test dataset (*n* = 5,062). On the x-axis, the reported ratios over the admin 1 and admin 0 levels are shown, whereas on the y-axis, the modelled ratio is shown.

After analysing the selected admin 1 level GDP ratio data for downscaling, we retained only the observations where the GDP ratio was less than or equal to 5 (i.e., we removed the outliers from the data). A rigorous search was conducted among some of the well-established machine learning algorithms to find the most suitable model for our purposes. After a preliminary analysis, multilayer perceptron (MLP)^31^, support vector regression (SVR)^32^, and ensembled trees^33^ were chosen for further study. The dataset (*n* = 25,307) consisted of coded data from 12 different meta-regions, following UN classification similar to that of Kummu *et al*.^34^. Eighty percent (80%) of the data from each of the 12 regions were randomly sampled to create the training set (*n* = 20,245). The remaining 20% of the data from each region were then used to create the testing set (*n* = 5,062), maintaining the balanced representation of regions in both sets. Each model has a set of hyperparameters to tune. To find the best hyperparameter sets for each model, we ran Bayesian optimisation^35^. We chose this method over grid search to avoid testing every possible configuration and tested only those that were more likely to improve the performance of the models. The ensembled trees model with 152 learners using the least-squares boosting method with a learning rate of 0.07 was chosen because it outperformed all the other models in both the training and testing phases (Fig. 3). Although we can see that the training accuracy for the ensemble method is much greater than the testing accuracy (Fig. 4) since the testing accuracy (R^2^ = 0.80) for this model is significantly greater than that of the runner-up MLP model (R^2^ = 0.49), it is still the superior model.

#### Downscaling error

**Fig. 5:**
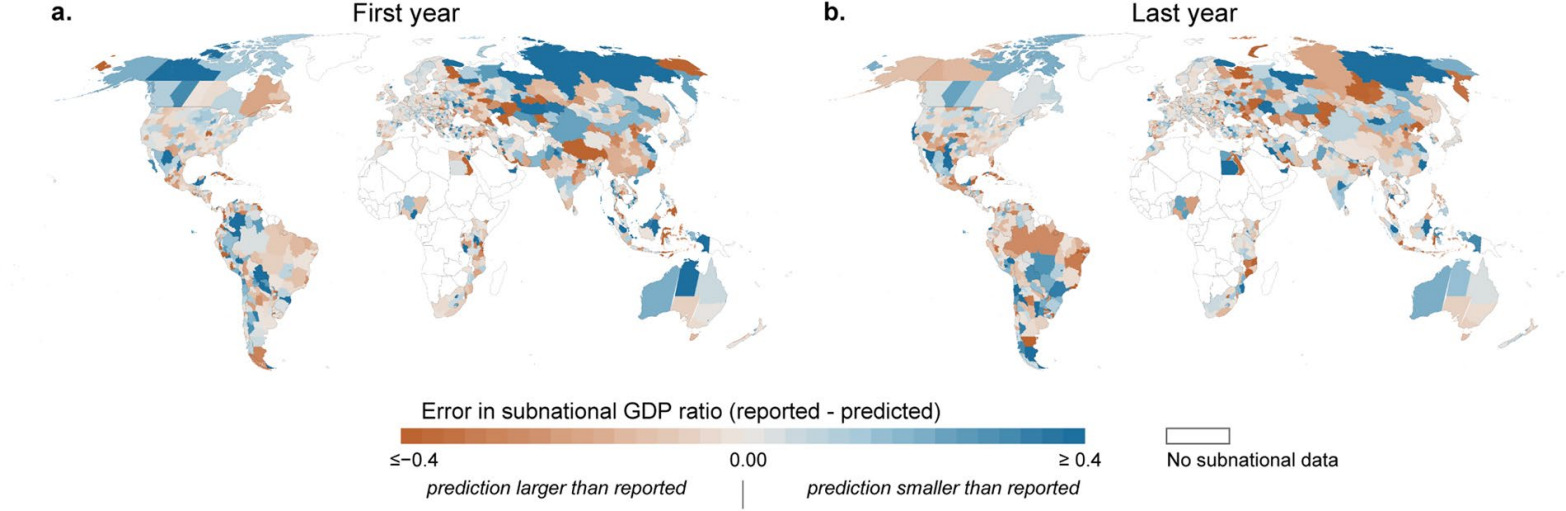
Downscaling model error of each administrative unit. Differences between reported and cross-validated predicted values for (**a**) first sampled year and (**b**) last sampled year.

To assess the downscaling error, we first mapped the subnational error (predicted subnational GDP ratio – reported subnational GDP ratio) for each admin unit (Fig. 5). We chose the first and last years sampled for training the downscaling model (years differ from country to country, see above), and it seems that the error is smaller the closer we are to the end of the study period. In general, the error is smallest in Europe and North America and largest in Central Asia and Latin America.

**Fig. 6:**
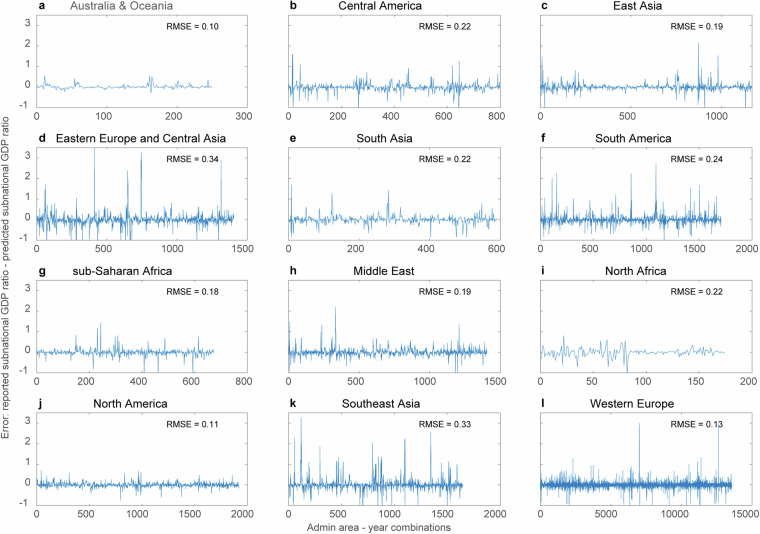
Downscaling error (reported–predicted) of the subnational GDP ratio for each administrative area‒year combination for 12 different meta-regions.

**Fig. 7:**
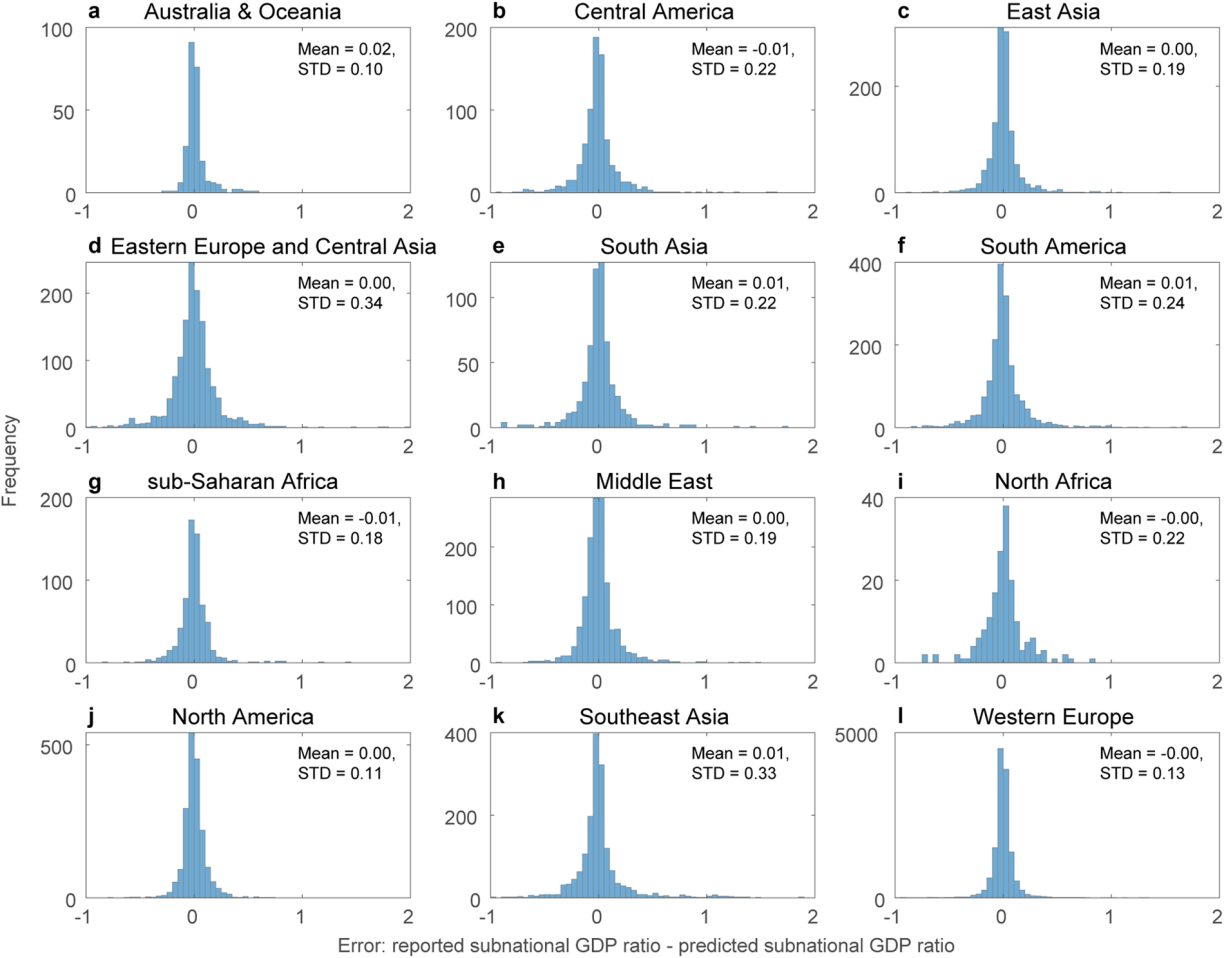
Error histogram of the downscaling model (reported – predicted) of the subnational GDP ratio for each of the 12 different meta-regions.

Furthermore, to assess the error in the downscaling method and how it differs from region to region, we used the 12 meta-regions (see the Downscaling procedure section) and plotted the error for each year-admin area combination (Fig. 6). These results show that, in general, the downscaling model predicts the subnational GDP ratio rather well, with the RMSE ranging from less than 0.15 (Australia & Oceania, North America and Europe) to 0.25-0.3 (Eastern Europe and Central Asia and Southeast Asia). Finally, we plotted the histogram for errors for each meta-region, as presented in Fig. 7.

### Prediction for the admin 2 level

To predict the admin 2 level GDP ratio (admin 2 level vs admin 1 level), we used the trained model with the independent variables aggregated to the admin 2 level. We obtained the administrative boundary layer for the admin 2 level from the GADM database (https://gadm.org). If there was no admin 2 level division for a country, we used the admin 1 level division instead. If this information was not available, then we used the admin 0 level division.

The data for the independent variables were the same as those used for training the downscaling model, with the exception that instead of using the admin 0 level data for the Gini coefficient^6^ and GDP per capita, we used here the admin 1 level data (i.e., again one administrative level higher than the administrative area in question). The validation for downscaled admin 2 level GDP per capita data is given in Section 4.

### Data harmonisation

To ensure consistency and comparability between subnational and national economic data (admin 1 and downscaled admin 2 levels), we implemented a data harmonisation procedure. The GDP per capita in purchasing power parity (PPP) was utilised to adjust for price level differences and variations in the cost of living between countries.

Subnational GDP per capita PPP data were collected from diverse sources and currencies, which we harmonised by calculating ratios relative to the national average (derived as the population-weighted average of the subnational data). This ratio-based approach, rather than using absolute values, was employed for interpolation, extrapolation, and downscaling. After downscaling, absolute subnational GDP per capita PPP values were computed based on the reported national figures.

To ensure further full alignment with national data, at the very end, we calculated the ratio between the population-weighted national average derived from subnational data (admin 1 level or admin 2 level) and the reported national data. This ratio was then used to correct biases in the subnational data. This detailed procedure, as depicted in Fig. 1, ensures comparable economic data across various regions that are harmonised with national reported data.

### Total GDP (PPP) data

We used GDP per capita Admin 2 level data to estimate total GDP (PPP) by multiplying the per capita data by the population count. We did this for three resolutions: 30 arc-sec (ca. 1 km at the equator), 5 arc-min, and 30 arc-min. To produce the 30 arc-sec product, we resampled the 5 arc-min GDP per capita data to that resolution.

For the 30 arc-sec product, we used GHS population grid (GHS-POP R2023A) data^26^ for each year for which population data were available (1990, 1995…, 2015, 2020). For the 5 arc-min product, we used the same GHS-POP R2023A population dataset (as with other parts of the dataset creation), aggregated to 5 arc-min resolution. Finally, we resampled the 5 arc-min data to 30 arc-min resolution.

## Data Records

The data are available at the following online repository: 10.5281/zenodo.10976733^36^. We provide the following data:

- GDP per capita (PPP) at the admin 0 level (national) for 1990–2022 (GeoTIFF, gpkg, csv)
- GDP per capita (PPP) at the admin 1 level (at the level of reporting, either the administration 1 level or the admin 0 level) for 1990–2022 (GeoTIFF, gpkg, csv)
- GDP per capita (PPP) at the admin 2 level (downscaled from the admin 1 level) for 1990–2022 (GeoTIFF, gpkg, csv)
- Total GDP (PPP), downscaled admin 2 level GDP per capita (PPP) multiplied by the gridded population count for 1990–2022, with three resolutions: 30 arc-sec, 5 arc-min, and 30 arc-min (GeoTIFF)
- The input data for the script were used to generate the data above (code_input_data.zip). The code is available at https://github.com/mattikummu/griddedGDPpc

The metadata with reported data years and sources for both the admin 0 and admin 1 levels are given in the Supplementary Material of this article.

**Fig. 8:**
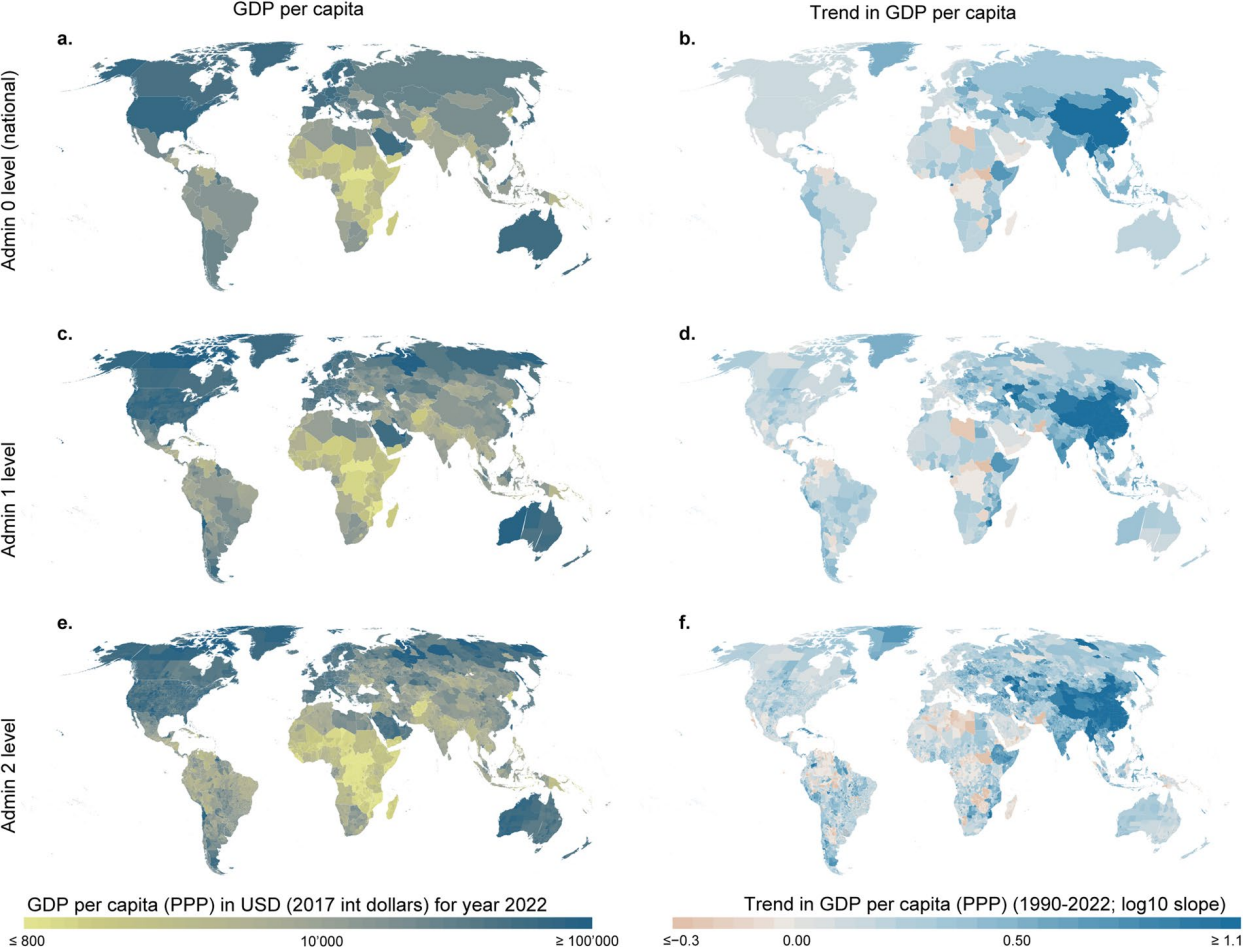
Comparison of the admin 0 level, admin 1 level and admin 2 level GDP per capita (PPP) products. (**a**) GDP per capita for the admin 0 level; (**b**) slope for the admin 0 level; (**c**) GDP per capita for the admin 1 level; (**d**) slope for the admin 1 level; (**e**) GDP per capita for the admin 2 level; and (**f**) slope for the admin 2 level. The GDP per capita is for 2022. Slope was estimated using the Siegel repeated medians method (calculated with R’s mblm package) over the study period (1990–2022). All maps are overlaid with country (admin 0 level) boundaries from Natural Earth data.

**Fig. 9:**
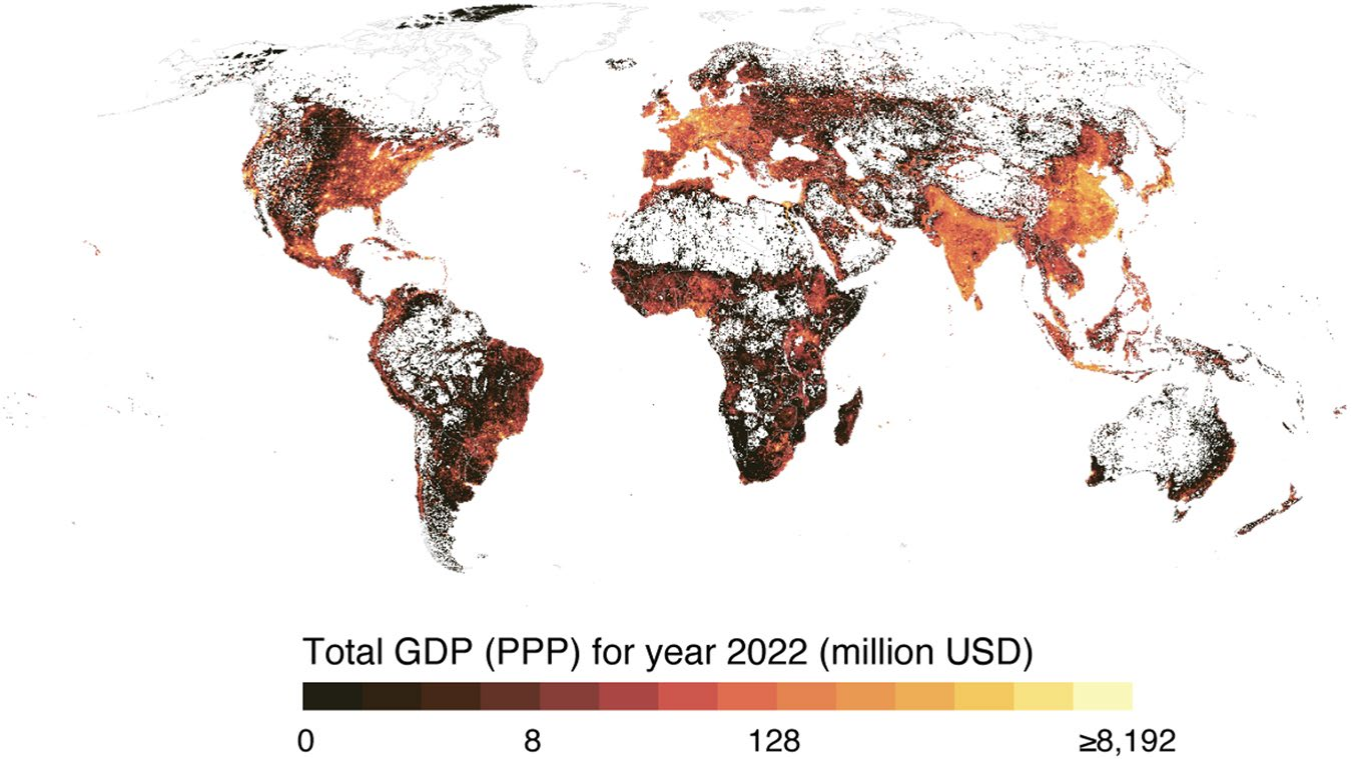
Total GDP (PPP) for 2022 in million USD. Plotted from the 5 arc-min resolution product, overlaid with country (admin 0 level) boundaries from Natural Earth data. Note: log2 scale for the colour scale.

The GDP per capita (PPP) data product for three administrative levels for 2022 and the slope over the study period of 1990–2022 using the Siegel repeated medians method (calculated with R’s mblm package) are shown in Fig. 8. The gridded total GDP (PPP) for 2022 is shown in Fig. 9.

## Technical Validation

We validated the final product at the admin2 level (downscaled from the admin 1 level) using subnational GDP per capita (PPP) data from OECD regional statistical databases (ref). The data include admin 2 level data for Belgium and Croatia (n = 43 subnational units), which were not used to create the dataset. These observed OECD data were compared against the aggregated downscaled GDP per capita (PPP) for the same administrative units.

**Fig. 10:**
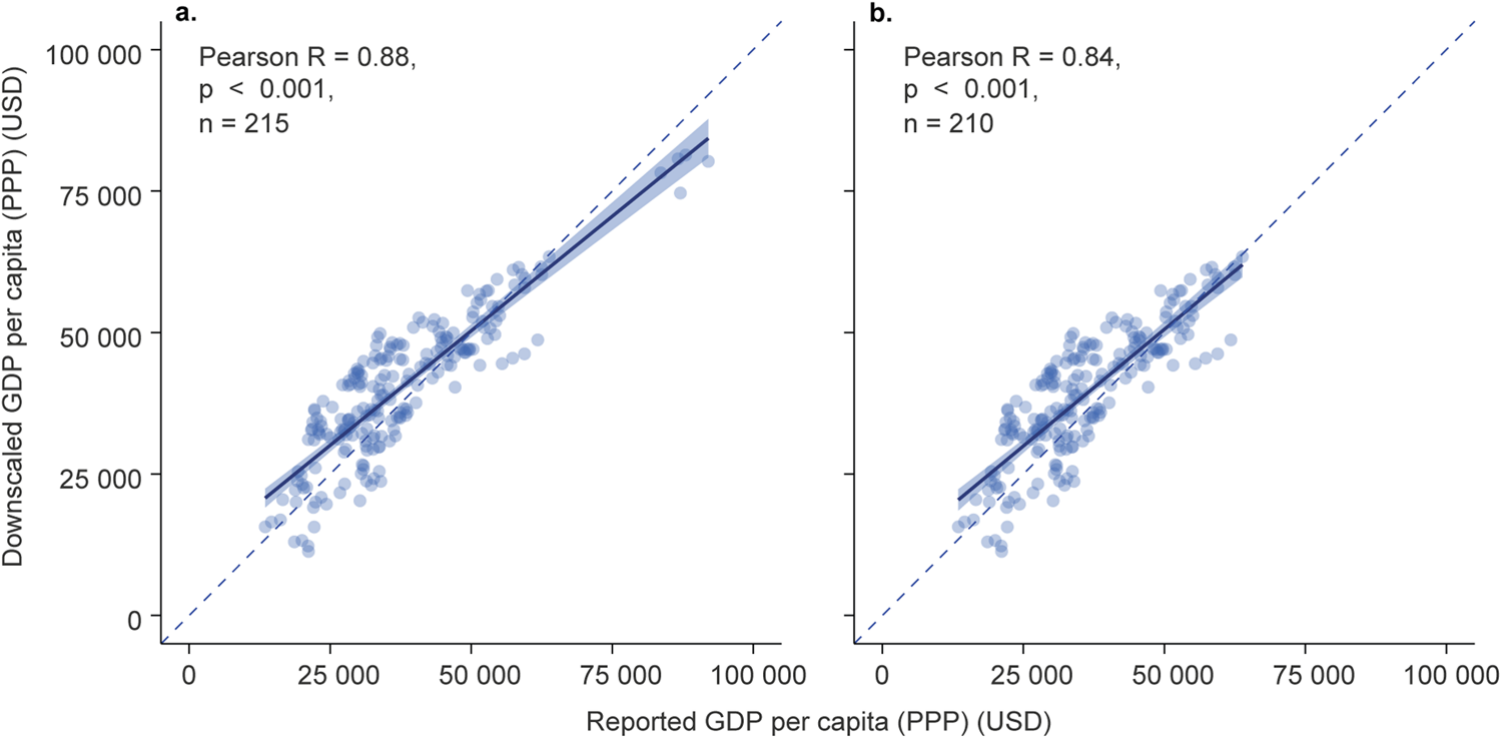
Validation of the downscaled admin 2 level data. Reported admin 2 level GDP per capita (PPP) for Belgium and Croatia compared against downscaled GDP per capita (PPP) for the same administrative areas. (**a**) all data; (**b**) the highest values were removed to test how they impact the correlation result. Correlation analysis was conducted with the Pearson correlation. The number of individual admin 2 level units is 43.

After aggregation, we harmonised the OECD subnational data with the national GDP per capita (PPP) data of our dataset and the OECD dataset. Then, we sampled 25% of the observed years and compared the downscaled values to the observed values using Pearson correlation (Fig. 10). The results show high accuracy, with a Pearson R of 0.88 (and without the highest GDP per capita points of 0.84).

**Fig. 11:**
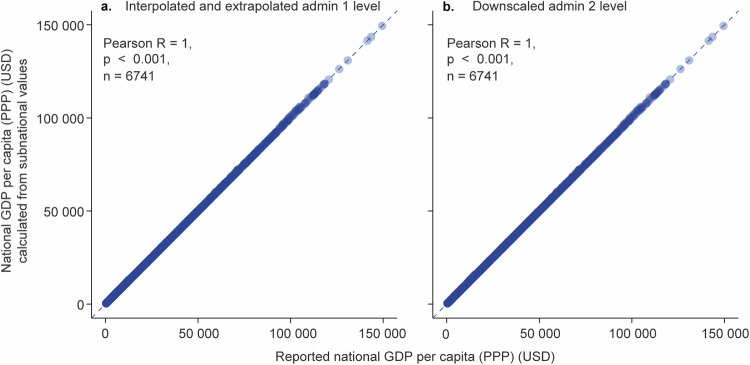
Validation of the aggregated admin 1 level (interpolated and extrapolated) and admin 2 level (downscaled) data against reported national level data. (**a**) aggregated admin 1 level data compared with reported national data; (**b**) aggregated admin 2 level data compared with reported national data. Correlation analysis was conducted with the Pearson correlation. Aggregation from subnational to national level was performed using the weighted population mean. The number of individual admin 0 level units is 235.

Furthermore, we validated how well our harmonisation of the subnational data products against reported national data by the World Bank, IMF and CIA worked. We did this by first aggregating the admin 1 level and admin 2 level data to the national level (admin 0 level) using population weighted average mean. We then compared these data with reported national-level data. We found that harmonisation had worked as intended, resulting in a Pearson’s R of 1 (p < 0.001) for both admin levels (Fig. 11).

## Usage Notes

**Fig. 12:**
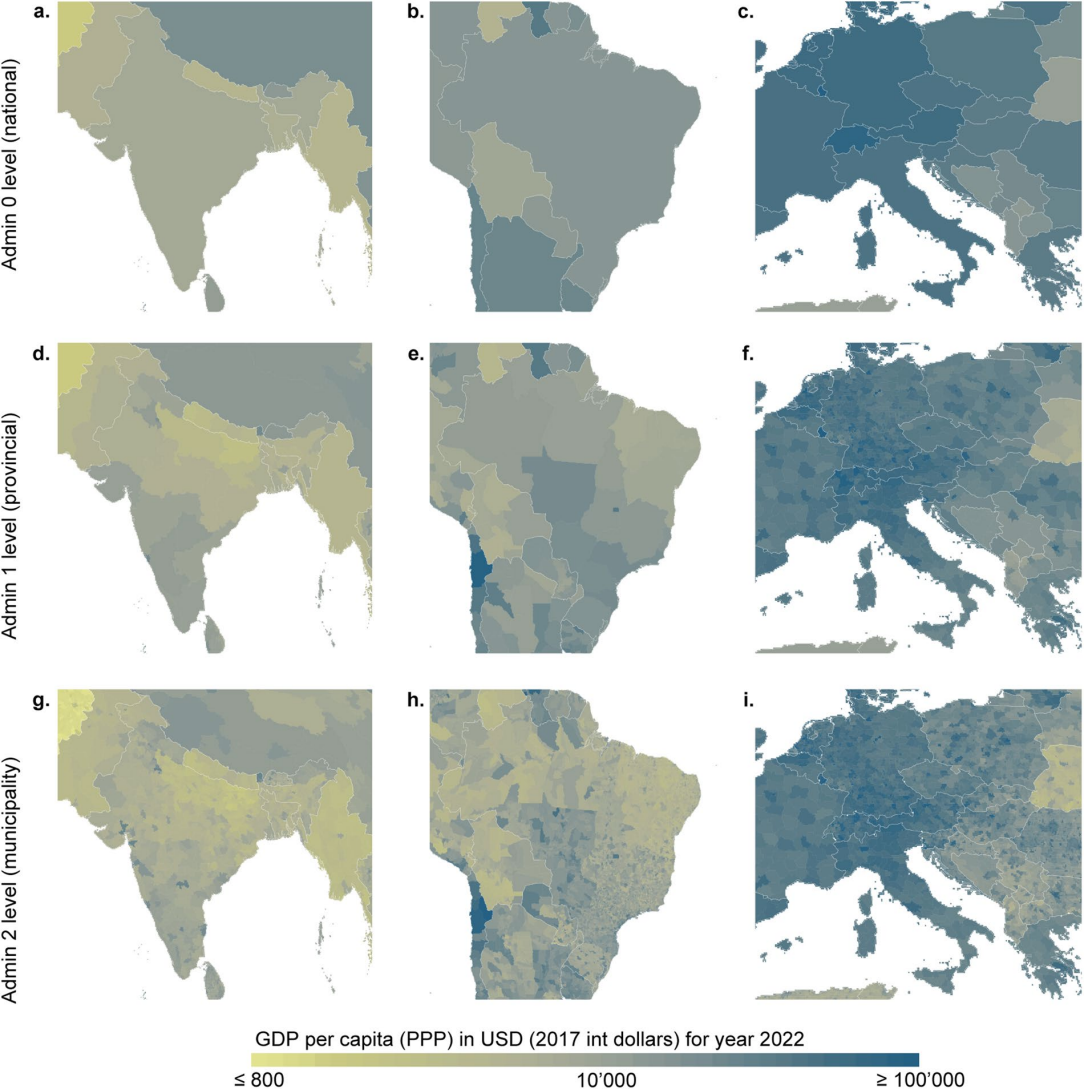
GDP per capita (PPP) for three different geographical areas. (**a,d,g**) South‒Southeast Asia; (**b,e,h**) Central South America; and (**c,f,i**) Central‒Southern Europe. (**a**–**c**) admin 0 level; (**d**–**f**) admin 1 level; and (**g**–**i**) admin 2 level. Plotted from the polygon GDP per capita (PPP) product, it is overlaid with country (admin 0 level) boundaries from Natural Earth data. Note: log10 scale for the colour scale.

**Fig. 13:**
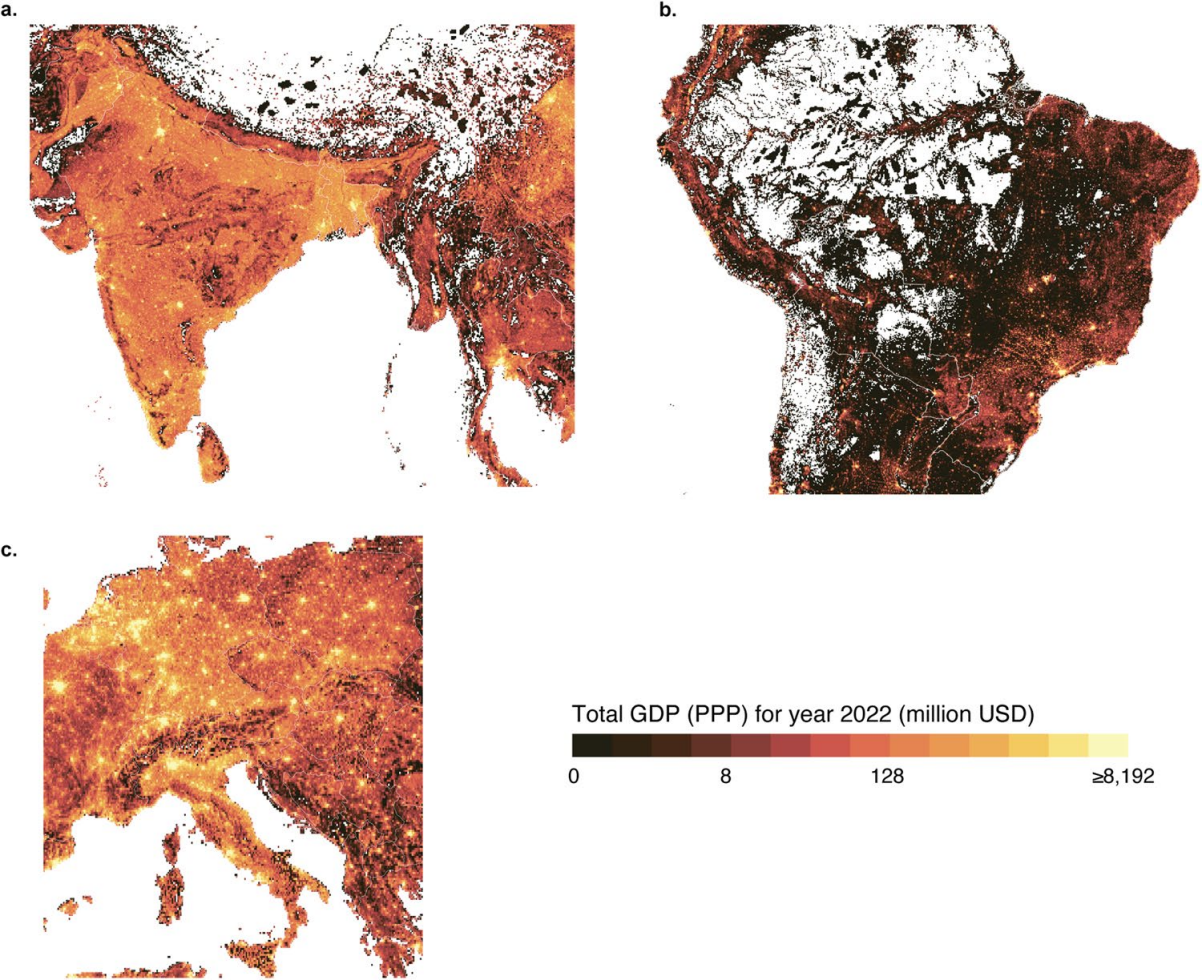
Total GDP (PPP) for 2020 in million USD for three geographical areas. (**a**) South‒Southeast Asia, (**b**) Central South America, (**c**) Central‒Southern Europe. Plotted from the 5 arc-min resolution product, overlaid with country (admin 0 level) boundaries from Natural Earth data. Note: log2 scale for the colour scale.

To show the usability of the data, we plotted the admin 0 level, admin 1 level and admin 2 level GDP (PPP) per capita data for three selected geographical areas (Fig. 12) for 2022. This demonstrates how much variability there is within shown countries and even within the 1-level administrative units. Furthermore, we also mapped the total GDP (PPP) for 2022 for the three selected geographical areas (Fig. 13), demonstrating better details than the results shown in a global map (Fig. 9).

## Supplementary information

Metadata for subnational data

## Data Availability

The code is available at the following github repository: https://github.com/mattikummu/griddedGDPpc. The input data for the code are available in the data repository: 10.5281/zenodo.10976733.
